# Biopsychosocial predictors of perceived life expectancy in a national sample of older men and women

**DOI:** 10.1371/journal.pone.0189245

**Published:** 2017-12-14

**Authors:** Lindsay C. Kobayashi, Rebecca J. Beeken, Susanne F. Meisel

**Affiliations:** 1 Harvard Center for Population and Development Studies, Harvard T. H. Chan School of Public Health, Cambridge, Massachusetts, United States of America; 2 Department of Behavioural Science and Health, University College London, London, United Kingdom; 3 Leeds Institute of Health Sciences, University of Leeds, Leeds, United Kingdom; 4 Institute of Psychiatry, Psychology & Neuroscience, King’s College London, London, United Kingdom; Nathan S Kline Institute, UNITED STATES

## Abstract

Perceived life expectancy (PLE) is predictive of mortality risk in older adults, but the factors that may contribute to mental conceptions of PLE are unknown. We aimed to describe the sociodemographic, biomedical, behavioral, and psychological predictors of self-reported PLE estimates among older English adults. Data were from 6662 adults aged 50–79 years in the population-based English Longitudinal Study of Ageing (cross-sectional sample from 2012/13). PLE was assessed in the face-to-face study interview (“*What are the chances you will live to be age x or more*?” where *x* = current age plus 10–15 years). Responses were categorized as ‘low’ (0–49%), ‘medium’ (50–74%), and ‘high’ (75–100%). Adjusted prevalence ratios (PRs) and 95% confidence intervals (CIs) for low vs. high PLE were estimated using population-weighted modified Poisson regression with robust error variance. Overall, 1208/6662 (18%) participants reported a low PLE, 2806/6662 (42%) reported a medium PLE, and 2648/6662 (40%) reported a high PLE. The predictors of reporting a low PLE included older age (PR = 1.64; 95% CI: 1.50–1.76 per 10 years), male sex (PR = 1.14; 95% CI: 1.02–1.26), being a smoker (PR = 1.39; 95% CI: 1.22–1.59 vs. never/former smoker), and having a diagnosis of cancer or diabetes. A low sense of control over life was associated with low PLE, as was low satisfaction with life and worse self-rated health. Those with a higher perceived social standing were less likely to report a low PLE (PR = 0.90; 95% CI: 0.87–0.93 per 10-point increase, out of 100). This study provides novel insight into potential influences on older adults’ expectations of their longevity, including aspects of psychological well-being. These results should be corroborated to better determine their implications for health-related decision-making, planning, and behavior among older adults.

## Introduction

Perceived life expectancy (PLE), the lifespan an individual anticipates to have remaining, is thought to have implications for health-related decision-making, planning, and behavior [1]. Many health-related decisions such as whether to participate in cancer screening, quit smoking, follow a particular treatment course for type II diabetes, or adhere to a physical exercise regime all represent investments in future health [2]. Such decisions may, in part, depend on how much ‘future’ one expects to have. This issue is of particular salience to older adults, who have highly variable and diminishing remaining life expectancies due to increasing age-related risks for chronic and acute conditions. Importantly, PLE is associated with mortality risk over two-year and eight-year periods among American men and women aged ≥50 years, indicating that older adults are reasonably good at assessing their remaining years [3]. With the aging structure of populations worldwide, whereby the number of adults over 80 is expected to triple to 434 million by 2050 from 125 million in 2015 [4], research on the influences upon PLE in older adults and the consequences of PLE for health-related outcomes is now more important than ever before.

To date, scientific interest in PLE has mainly been in relation to its effect on economic decision-making and planning, such as retirement transitions and financial savings [5–8], although there is a small and growing body of evidence associating PLE with health-related outcomes. In a sample of 370 adults who had been ‘prescribed’ exercise for rehabilitation, Ziegelmann and colleagues showed that those with low longevity expectations at baseline were less likely to adhere to exercise recommendations over a one-year follow-up [9]. Cross-sectionally, PLE has been associated with smoking status and alcohol consumption in men, attendance at mammography screening in women, but has been inconsistently associated with diet and exercise in men and women [10–12]. Prospectively, PLE has been shown to independently predict long-term patterns of physical activity and smoking, and participation in colorectal cancer screening among older adults [13,14]. Although further research is required to assess whether PLE is causally related to mortality over and above its utility as an individual estimate of personal longevity, it may influence mortality risk through these suggested effects on health-related behaviors.

While only these few studies have investigated PLE in relation to health behaviors and other outcomes, additional evidence in this body of literature comes from research on the psychological construct of time perspective (i.e. one’s preference for the past, present, or future). A limited future time perspective is thought to have a negative effect on the planning and execution of health-promoting behaviors such as physical activity and cancer screening, which involve short-term discomfort or costs for long-term benefits to health [15,16]. A future-oriented time perspective has been positively associated with health behaviors such as participation in cancer screening, smoking cessation, better coping with HIV management, and greater physical activity and fruit and vegetable consumption [16–21]. Although PLE is not the same concept as time perspective, and there is a dearth of studies directly comparing these two measures, older adults with a low PLE may also often have a limited future time perspective due to their perceived limited future lifespan.

PLE therefore may influence the timing of an individual’s life transitions and future long-term patterns of their health-related behaviors, which would contribute to patterns of population health and health care system use. However, beyond one’s current age and the ages of one’s parents (either currently or at death), the factors upon which older people may base their expectations of remaining life expectancy are not well understood [3,10]. Griffin et al proposed a comprehensive theoretical model using a biopsychosocial framework of influences on PLE [10]. The underlying premise of the model is that since PLE estimates tend to be reasonably accurate when compared to actual mortality rates, the factors influencing PLE should be similar to actual mortality risk factors [10]. The model also draws upon the notion that individuals develop internal representations about their likely ages at death based on their interpretations of their personal experiences and contexts [22]. Therefore, the model includes *biomedical factors* (age, sex, age of parents, and number of health conditions), *socioeconomic factors* (income and education), *health behaviors* (body mass index, smoking status, alcohol consumption, physical activity, and diet), and *psychosocial factors* (optimism, psychological distress, and social connectedness) [10].

When empirically tested in a population-based sample of 2759 Australian adults aged 57 to 67 years who were in paid work at the time, the statistically significant factors in the model were age, age of parents, body mass index, smoking (in men), optimism, psychological distress (in women), and social connectedness (in men) [10]. The three latter factors were the most strongly associated with PLE, over and above the biomedical and behavioral factors. Psychological indicators of well-being are associated with all-cause mortality risk in older adults [23], although little is known about their relationship with PLE aside from Griffin et al.’s testing of their theoretical model [10]. If there truly is an association between psychological states and PLE, their potential modifiability might make them good candidates for interventions to help improve low expectations of the future, and in turn, improve health-related decision-making, planning, and behavior among older adults.

This biopsychosocial model of PLE requires testing in other study samples, particularly among elderly adults, and there are areas where it deserves to be improved upon. For example, income can be a poor economic measure for older adults, who are often retired or have different sources of earnings than working-age adults [24]. Rather, measures of total wealth adjusted for retirement status are better economic indicators for older populations. Further, Griffin’s original model operationalized health status as the sum total of health conditions a person had been diagnosed with, rather than individual conditions such as heart disease, diabetes, or cancer, which are associated with differing risks for mortality. Finally, the dominance of the three psychosocial factors in the model’s empirical testing indicated that psychological factors might be a key influence over how people develop PLE estimations. If this were the case, then perceptions about social situations might matter for more PLE than objectively measurable indicators of social position. For instance, feeling lonely or feeling that one is poor might be more important than living alone or being poor for informing PLE estimations [24]. Indeed, previous work shows that among older adults, perceived social status is a robust predictor of health outcomes that reflects personal realities internalized from prevailing socioeconomic conditions [24].

In this paper, we utilize Griffin et al.’s biopsychosocial model of PLE, refining and expanding upon it with additional objectively measured socioeconomic and health status indicators and subjectively measured psychological factors that capture internalized life circumstances. Drawing upon the notion that people utilize knowledge of their current and recent health and behavior to form expectations of their future longevity [3,10,22], we utilize a cross-sectional sample where predictor variables and PLE were assessed in a single data collection episode. The objective of this study was to investigate the sociodemographic, biomedical, health condition, health behavior, and psychological factors associated with perceived life expectancy in a population-based sample of English men and women aged 50 to 79 years.

## Materials and methods

### The English longitudinal study of ageing

Data were from Wave 6 (2012–13) of the English Longitudinal Study of Ageing (ELSA), a prospective cohort of English adults aged ≥50 years [25]. The cohort was established in 2002, based on a random stratified sample of English households (n = 12 100) [26]. All ELSA participants who completed data collection at Wave 6 and were aged 50–79 years with non-proxy interviews were eligible for this analysis (n = 7909).

### Ethical approval

The ELSA was approved by the London Multicentre Research Ethics Committee (MREC/01/2/91) and informed consent was obtained from all participants. All procedures were conducted in accordance with the ethical standards of the responsible committee on human experimentation (institutional and national) and with the Helsinki Declaration of 1975, as revised in 2000.

### Perceived life expectancy

PLE was assessed as part of the in-person study interview with the question, “What are the chances you will live to be age *x* or more?” The variable *x* was defined as follows: if the participant was age 65 or under, *x* = 75; if age 66–69, *x* = 80; if age 70–74, *x* = 85; if age 75–79, *x* = 90; if age 80–84, *x* = 95; if age 85–99, *x* = 100; if age 100–104, then *x* = 105; if age 105–109, *x* = 110, if age 110–119, *x* = 120. The participant was presented with a show card with a scale from 0 to 100 to aid in his or her response.

PLE is a construct that tends to generate modal focal responses in research studies, which is a type of cognitive bias where respondents will round their answer to values such as 0, 50, and 100 when responding on a 0 to 100-point scale, rather than responding on a truly continuous scale [3,12,27]. In reality, life expectancy lies along a continuous scale without modal probabilities. Responses of 0% and 100% are agreed to represent misunderstanding of risk and probabilities, given that absolute certainty of an outcome such as mortality is nearly impossible to predict [7,27,28]. These extreme modal responses can be smoothed through categorization of responses at the upper and lower ends of the 0 to 100 scale [12]. Other focal points are thought to represent real probabilities that lie close to the focal point in question, to which the respondent has rounded his or her response [28].

**Fig 1:**
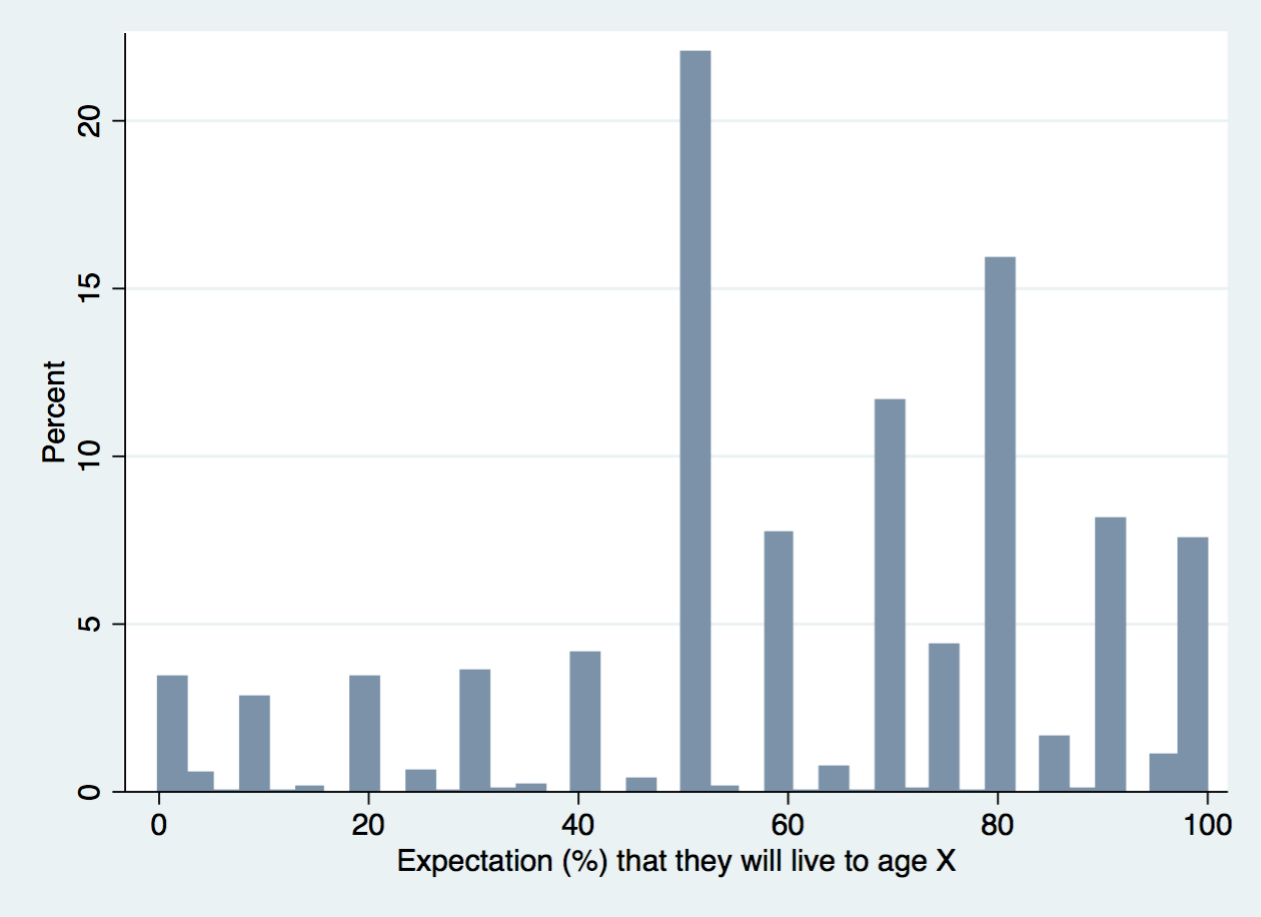
Distribution of responses to the perceived life expectancy interview question (%).

In our sample, 7799/7909 (98.6%) eligible participants responded to the life expectancy question. Of these, 257 (3.3%) responded with a probability of 0% (0.0) and 567 (7.3%) responded with 100% (1.0). More common focal responses in our sample were 50% (1716; 22.0%), 70% (906; 11.6%), and 80% (1237; 15.9%). Fig 1 shows the full distribution of responses. To smooth out any focal response errors, we categorized PLE as a three-category variable of 0–49%, 50–74%, and 75–100% [26]. As only 19% of the sample was in the 0–49% range, this category was not further split. There was strong correspondence between the PLE estimates and all-cause 10-year mortality risk according the ELSA Mortality Risk Index (Table A in S1 File) [29].

### Predictors of PLE

For consistency with a theoretical biopsychosocial model of PLE among older adults [10], we defined four domains of potential predictor variables: sociodemographic factors, biomedical factors, health behaviors, and psychological factors. The variables we selected within each category have either been empirically shown to be associated with PLE, or are plausibly associated with PLE based on the biopsychosocial model [10]. We include additional socioeconomic and health indicators, as well as psychological factors, which are associated with a range of self-reported and clinical health measures and all-cause mortality risk among older adults [23,25]. The included variables are as follows:

#### Sociodemographic factors

Educational attainment (higher education; intermediate-level education; no qualifications); net non-pension wealth in quintiles (calculated stratified at age 65 to account for the effect of retirement on wealth); and marital status (married or cohabiting; single).

#### Biomedical factors

Current age (per 10 years); age at which mother died or current age (per 10 years); age at which father died or current age (per 10 years); sex (male; female); presence of depressive symptoms (>3 out of 8 symptoms) [30]; and self-reported physician diagnosis of cardiovascular disease (angina, heart attack, or stroke), cancer, diabetes, or chronic lung disease (all yes vs. no). Body mass index (BMI) was measured for a subset of ELSA participants who agreed to a nurse visit during their study interview (continuous and stratified at <30 vs. ≥30 to indicate obesity).

#### Health behaviors

Current smoking status (smoking vs. not smoking); drinking status in the past year (abstainer; non-excessive drinker [less than five days per week]; excessive drinker [five or more days per week]); daily fruit and/or vegetable intake (<5 vs. ≥5 servings per day); and weekly engagement in physical activity (none/mild vs. moderate to vigorous). These categories were selected for each variable because they reflect UK public health guidelines for lifestyle behaviors and represent biologically meaningful thresholds associated with increased risks of chronic disease and mortality, and [31–36].

#### Psychological factors

Scoring quartile for each domain of the CASP-19, a validated 19-item measure of quality of life that encompasses domains of life control (C), autonomy (A), life satisfaction (S), and pleasure (P); loneliness according to the 3-item revised UCLA scale; and, perceived social status (per 10 points out of 100 possible). The CASP-19 asks questions such as, ‘I feel that what happens to me is out of my control’ (C), ‘I can do the things that I want to do’ (A), ‘I feel satisfied with the way my life has turned out’ (S), and ‘I look forward to each day’ (P), with response options of ‘never’, ‘not often’, ‘sometimes’, and ‘often’ [37]. The UCLA loneliness scale asks participants questions such as ‘how often do you feel lonely?’ with response options of ‘hardly ever or never’, ‘some of the time’, and ‘often’ [38]. Perceived social status was assessed with an image of a ladder with ten rungs [25]. Participants were asked to imagine the ladder representing society, where those best off were at the top of the ladder and those worst off on the bottom, and to mark a cross on the rung where they would place themselves in society. The ladder was scored from 0–100 (10 points per rung). Self-rated health, which captures aspects of both physical health and psychological well-being [39], was measured as ‘excellent’, ‘very good’, ‘good’, ‘fair’, and ‘poor’.

### Statistical analysis

All predictor variables were examined bivariately against the PLE variable, using the chi-squared test for categorical variables and Student’s t-test for continuous variables. Correlations between all of the predictor variables were calculated. A population-weighted and multivariable-adjusted modified Poisson regression model with robust error variance for binary outcome data was used to estimate prevalence ratios (PRs) and 95% confidence intervals (CIs) for each of 0–49% and 50–74% vs. 75–100% perceived probability of survival [40]. These two levels of the outcome were predicted separately because the proportional odds assumption was violated when an ordinal regression model was tested. All predictor variables that were associated with PLE with *p*<0.10 in bivariate analysis were included in the model. Net non-pension wealth and BMI were trimmed from the model, as these two variables were missing data on >5% of observations and they were non-statistically significant in the model with their removal not altering other effect estimates by ≥10% [41]. Population weights that adjusted for differences in the propensity to respond amongst key population subgroups were applied to all models. The population weights were calculated at each data collection wave of ELSA, iteratively adjusting for non-response at all waves using data from the UK 2001 Census to characterize the target general population [42]. Tolerance and variance of inflation factors (VIF) were calculated to assess collinearity in the model, with tolerance values of <0.10 and VIF values of >10 indicating possible collinearity [43]. All analyses were conducted at the 95% confidence level using StataSE 13.1 (College Station, TX).

### Sensitivity analyses

We conducted two sensitivity analyses. The first excluded responses of 0% (n = 198) and 100% (n = 471) to assess whether the respondents who made these particular focal point errors were misclassified in a way that affected the results. The second was an exploratory re-analysis of the model stratified by age at <65 vs. ≥65 years. This was done to assess whether age might modify responses to the PLE question, as the expectation to live another 10–15 years might mean different things to respondents at either end of the age range in this study. The continuous variable for age was coded per year in the stratified model rather than per 10 years as in the full sample model, otherwise all variables were the same as in the full sample model.

## Results

### Missing data and final sample size

Of the eligible 7909 participants, 110 (1.4%) did not respond to the life expectancy question and were excluded. Of the remaining 7799 eligible participants, 1137 (15%) were missing data on at least one predictor variable, although less than 5% of data were missing for any single variable. The final multivariable model predicting PLE therefore included a total of 6662 participants (85% of eligible). Missing data are summarized and the characteristics of participants with missing data are presented in Table B in S1 File.

### Sample characteristics

Participant characteristics are shown in Table 1. Overall, 1208/6662 participants reported a low PLE (18%), 2806/6662 reported a medium PLE (42%), and 2648/6662 reported a high PLE (40%). One-third of participants reported having a higher education degree (35%; 2310/6662), while one-fifth reported having no educational qualifications (20%; 1309/6662). Most were married or cohabiting (77%; 5132/6662) and just under half were male (45%; 3006/6662). The mean age was 64.5 years (standard deviation [SD]: 7.4 years). The mean reported age of mother currently or at death was 77.8 years (SD: 12.6 years), and mean reported age of father at currently or at death was 72.8 years (SD: 13.2 years). The biomedical, health behavioral, and psychological characteristics of participants are also shown in Table 1. The correlations between all variables were of moderate to weak strength (Table C in S1 File). The tolerance and VIF values revealed no collinearity between variables (Table D in S1 File).

**Table 1:** Participant characteristics, the English longitudinal study of ageing, 2012–13 (n = 6662).

Participant Characteristics	N (%)
Perceived life expectancy	
0–49%	1208 (18%)
50–74%	2806 (42%)
75–100%	2648 (40%)
**Sociodemographic factors**	
Educational attainment	
Higher education	2310 (35%)
Intermediate	3043 (46%)
No qualifications	1309 (20%)
Wealth quintile^a^	
5 (richest)	1403 (22%)
4	1365 (22%)
3	1294 (21%)
2	1218 (19%)
1 (poorest)	1002 (16%)
Married or cohabiting^a^	5132 (77%)
**Biomedical factors**	
Age (mean; SD)	64.5 (7.4)
Mother’s age at death (mean; SD)	77.8 (12.6)
Father’s age at death (mean; SD)	72.8 (13.2)
Male sex	3006 (45%)
Depressive symptoms^a^	1436 (22%)
Cardiovascular disease^a^	604 (9%)
Cancer^a^	242 (4%)
Diabetes^a^	668 (10%)
Chronic lung disease^a^	279 (4%)
BMI (mean; SD)	28.3 (5.2%)
BMI ≥30	1708 (31%)
**Health behaviors**	
Current smoking	785 (12%)
Alcohol consumption	
Abstainer	777 (12%)
Not excessive	4420 (66%)
Excessive	1465 (22%)
Fruit & vegetable intake <5 per day	2746 (41%)
Physical activity	
None or mild	1166 (17%)
Moderate-to-vigorous	5496 (83%)
**Psychological factors**	
Loneliness (max 9; mean; SD)^a^	4.0 (1.2)
Control (max 12; mean; SD) ^a^	8.0 (2.4)
Autonomy (max 15; mean; SD) ^a^	10.2 (2.7)
Life satisfaction (max 15; mean; SD) ^a^	10.1 (3.1)
Pleasure (max 15; mean; SD) ^a^	13.1 (2.3)
Perceived social status (max 100; mean; SD) ^a^	59.0 (17.2)
Self-rated health	
Poor	402 (6%)
Fair	1128 (17%)
Good	2118 (32%)
Very good	2097 (32%)
Excellent	917 (14%)

^a^These variables are in addition to those originally included in Griffin et al.’s biopsychosocial model of PLE [10]

### Sociodemographic factors

Educational attainment and marital/cohabiting status were not associated with PLE (Table 2).

**Table 2:** Population-weighted and adjusted prevalence ratios (PRs) for medium and low vs. high perceived life expectancy (PLE) associated with sociodemographic, biomedical, behavioral, and psychosocial factors, the English longitudinal study of ageing, 2012–13 (n = 6662).

Characteristic	PR^a^ Medium PLE	95% CI	PR^b^ Low PLE	95% CI
**Sociodemographic factors**				
Intermediate education (vs. higher)	1.03	(0.95, 1.11)	1.04	(0.91, 1.19)
No educational qualifications (vs. higher)	1.03	(0.94, 1.13)	1.10	(0.95, 1.27)
Single marital status^c^	1.05	(0.91, 1.21)	1.01	(0.90, 1.13)
**Biomedical factors**				
Age (per 10 years)	1.16	(1.10, 1.22)	1.64	(1.50, 1.78)
Mother’s age at death (per 10 years)	0.95	(0.93, 0.97)	0.91	(0.88, 0.94)
Father’s age at death (per 10 years)	0.97	(0.95, 0.99)	0.92	(0.89, 0.95)
Male sex	1.03	(0.96, 1.10)	1.14	(1.02, 1.26)
Depressive symptoms^c^	0.97	(0.89, 1.06)	0.97	(0.86, 1.09)
Cardiovascular disease^c^	0.88	(0.79, 0.98)	0.98	(0.86, 1.11)
Cancer^c^	1.25	(1.07, 1.45)	1.24	(1.03, 1.49)
Diabetes^c^	1.00	(0.89, 1.11)	1.11	(0.98, 1.25)
Chronic lung condition^c^	1.08	(0.94, 1.25)	1.06	(0.92, 1.23)
**Health behaviors**				
Current smoking	1.13	(1.03, 1.24)	1.39	(1.22, 1.59)
Any MVPA (vs. mild/no physical activity)	1.14	(1.05, 1.25)	1.15	(1.01, 1.32)
Less than five daily fruit or vegetable servings	1.06	(0.99, 1.13)	1.06	(0.95, 1.18)
Non-excessive alcohol drink (vs. abstainer)	0.95	(0.88, 1.02)	0.99	(0.84, 1.14)
Excessive alcohol drinker (vs. abstainer)	0.85	(0.73, 0.99)	1.00	(0.95, 1.18)
**Psychological factors**				
Loneliness^c^^,^^d^	0.96	(0.93, 0.99)	0.94	(0.90, 0.99)
Control^c^^,^^d^	0.99	(0.97, 1.01)	0.96	(0.93, 0.99)
Autonomy^c^^,^^d^	1.01	(0.97, 1.05)	0.94	(0.88, 1.01)
Pleasure^c^^,^^d^	0.99	(0.94, 1.05)	0.99	(0.91, 1.08)
Life satisfaction^c^^,^^d^	0.91	(0.87, 0.95)	0.91	(0.84, 0.98)
Perceived social status (per 10 points)^c^	0.97	(0.95, 0.99)	0.91	(0.88, 0.94)
Self-rated health^d^	0.89	(0.86, 0.93)	0.75	(0.70, 0.80)

^a^PR predicts medium (50–74%) vs. high (75–100%) PLE

^b^PR predicts low (0–49%) vs. high (75–100%) PLE

^c^These variables are in addition to those originally included in Griffin et al.’s biopsychosocial model of PLE [10]

^d^PR is for increasing linear trend from the lowest to highest quartiles of the variable

### Biomedical factors

For each 10-year increase in age, the PR for reporting a medium PLE increased by 16% (PR = 1.16; 95% CI: 1.10–1.22) and the PR for reporting a low PLE increased by 64% (PR = 1.64; 95% CI: 1.50–1.78; Table 2). Ages of mother and father currently or at death were also associated with PLE, where those with an older age of a parent at death were less likely to report a medium or low PLE (Table 2). Men more often reported a low PLE than women (PR = 1.14; 95% CI: 1.02–1.26; Table 2). Depressive symptoms, cardiovascular disease, and chronic lung conditions were not associated with PLE. A diagnosis of cancer was associated with reporting a medium or a low PLE (PR = 1.24; 95% CI: 1.03–1.49 for low PLE; Table 2). Having diabetes was associated with reporting a low PLE, although the lower limit of the 95% CI just crossed the null (PR = 1.11; 95% CI: 0.98–1.25).

### Health behaviors

Current smokers were more likely than former or non-smokers to report a medium or PLE (PR = 1.13; 95% CI: 1.03–1.24 for medium PLE; PR = 1.39; 95% CI: 1.22–1.59). Engagement in weekly moderate-to-vigorous physical activity was associated with reporting a medium (PR = 1.14; 95% CI: 1.05–1.25) or low PLE (PR = 1.15; 95% CI: 1.01–1.32; Table 2). Excessive alcohol consumption (vs. non-excessive) was associated with reduced likelihood of reporting a medium PLE (PR = 0.85; 95% CI: 0.73–0.99), although all other categories of alcohol consumption were not associated with PLE (Table 2).

### Psychological factors

Loneliness was inversely associated with PLE, with PR = 0.94 (95% CI: 0.93–0.99) for low PLE with increasing loneliness quartile (Table 2). A higher sense of control over life was also inversely associated with reporting a low PLE in a dose-response fashion, with PR = 0.96 (95% CI: 95% CI: 0.93–0.99) per increasing quartile of control. Life satisfaction was associated with low PLE in a dose-response fashion, with PR = 0.91 (95% CI: 0.84–0.98) per increasing quartile. The other two domains of the CASP-19, autonomy and pleasure, were not independently associated with PLE. Adults with a higher perceived social status were less likely to report a medium or low PLE (PR = 0.91; 95% CI: 0.88–0.94 for low vs. high PLE, for every 10-point increase out of 100). Self-rated health was inversely associated with low PLE, with PR = 0.75 (95% CI: 0.70–80) per each increasing level of self-rated health from ‘poor’ to ‘excellent’ (Table 2).

### Sensitivity analyses

When respondents who gave themselves a 0% or 100% chance of living another 10–15 years were excluded from the analysis, the results of the study were nearly identical to the original analysis. When the model shown in Table 2 was stratified by age (<65 years vs. ≥65), there were a few small, non-significant differences in effect estimates (Tables E and F in S1 File). In general, the PRs for chronic diseases were stronger among the <65 age group, and the PRs for the health behaviors were stronger in the ≥65 age group, but the CIs overlapped for the PRs between the two groups (Tables E and F in S1 File).

## Discussion

In this large, population-based sample of men and women aged 50 to 79 years in England, we found that the cross-sectional predictors of perceived life expectancy were consistent with actual risk factors for mortality among older adults [29]. These predictors included current chronological age, age of parents currently or at death, gender, smoking status, moderate-to-vigorous physical activity, self-rated health, and the presence of chronic health conditions such as cancer. In addition, we identified novel psychological predictors of PLE: sense of control over one’s life, life satisfaction, and perceived social status. These results underscore the importance of understanding people’s expectations of future health in later life, building upon recent literature linking psychological well-being to health and longevity [23,44–47]. The improvement of psychological well-being among older adults may therefore have the benefit of improving life expectancy perceptions. Our results should be corroborated in other studies. Further, the predictors of longitudinal within-person changes in PLE, and effects of PLE on health-related behaviors and outcomes should be investigated.

### Comparison to existing literature

Although there is little research in this area, our results are consistent with Griffin et al.’s study that tested predictors of PLE from their theoretical biopsychosocial model [10], as well as data from the baseline 2002–03 wave of ELSA that validated PLE estimates against observed mortality over a six-year follow-up [48]. Consistent with Griffin et al., we found that education was not associated with PLE; neither was net non-pension wealth in our study or annual household income in their study. Older current age and older age of parents currently or at death and smoking status were strong predictors of PLE in both their study and ours [10]. BMI and exercise were not associated with PLE in Griffin et al.’s study, and we also observed no association with BMI but an inverse relationship between exercise and PLE [10]. In our study, men were more likely than women to report a low PLE, although Griffin et al. observed no gender differences. Griffin et al. did not find that the sum total of 13 different health conditions was associated with PLE, but we found that individual diagnoses of cancer and diabetes were associated with reporting a low PLE. Some of these divergent findings may be due to the different measures of PLE used in each study (Griffin et al. asked respondents to report what age they think they will live to; we asked respondents what they think is the percent chance they will live another 10–15 years), as well as different operationalizations of the predictor variables, and differences in sample composition. Finally, in both studies, psychological measures were strong predictors of PLE (optimism and distress in Griffin et al.; control over life, life satisfaction, and perceived social standing in our study), indicating that psychological state at the time of measurement may be an important influence on mental conceptions of perceived life expectancy.

We newly identified that a low sense of control over life, low life satisfaction, and low perceived social standing were associated with worse PLE in older adults in England. These factors are associated with risk of all-cause mortality among older adults [23]. Their potential influence on expectations of longevity could result in engagement in negative health-related behaviors [13], and the subsequent development of health conditions that would raise mortality risk. Improvement of global quality of life, including sense of control over life and life satisfaction, could therefore be a target for system-level interventions and policies seeking to improve health outcomes for older adults. Empowerment interventions have proven to be successful for improving health and well-being in patient samples, but have not typically been used with ‘healthy’ populations [49,50]. Health and social policies also have key roles to play in ensuring that older adults have the opportunities necessary to lead fulfilling lives.

Smoking and moderate-to-vigorous physical activity, but not fruit and vegetable intake, BMI, or alcohol consumption in the past year were associated with PLE. These results are consistent with those produced by Adams et al., who used cross-sectional data from the baseline 2002–03 wave of the ELSA showing that PLE is predictive of smoking and physical activity, but not alcohol consumption [13]. Adams et al. also investigated longitudinal relationships, whereby low PLE at baseline was predictive of taking up or relapsing smoking and of declining in physical activity levels over an eight-year period [13]. However, their findings may be confounded as they were not adjusted for health status or chronic conditions aside from limitations in activities of daily living. Their study hypothesized the reverse direction of association to our study, which is entirely plausible. Bidirectional relationships may exist between expectations of future longevity and health behaviors among older adults, and they require further investigation. Our results are also consistent with a study showing that baseline smoking status, but not baseline diet or BMI were prospectively associated with PLE as an outcome after a three-year follow-up [10]. However, diet and exercise have been positively associated with PLE in a cross-sectional study [11]. The causality of relationships between health behaviors and PLE, the duration of time between engagement in a behavior and its potential influence on PLE, and whether or not bidirectional relationships might exist between behavior and PLE, require further investigation. If low PLE is perceived as an uncontrollable threat to health that would negatively affect future health-related behaviors, as indicated in two recent longitudinal studies of older adults [13,14], then health practitioners should take this barrier into account when giving advice to older patients.

While educational attainment was not significantly associated with PLE in our study, other evidence shows that adults with low socioeconomic status often downgrade or inaccurately estimate their remaining life expectancy [48,51,52]. Research using the baseline 2002–03 wave of the ELSA found that older adults and those with lower income were more inaccurate in their PLE estimates than younger and wealthier adults, when PLE estimates were validated against six-year mortality data [48]. A similar phenomenon has been observed with self-rated health, where socioeconomically disadvantaged adults in the U.S. were more likely to downgrade their health than more advantaged adults [53]. Given that PLE appears predictive of future health behaviors and mortality risk, then lowered expectations of future longevity among socioeconomically deprived adults, especially if inaccurate, may contribute to social inequalities in health. This issue deserves more attention in public health research and interventions.

### Limitations and strengths

An important caveat of this study is that we do not know whether or to what degree participants may have taken the factors we investigated into account when responding to the study interview question on PLE. Consequently, some associations may be due to residual confounding. An example is if having a poor self-rated health is associated with an unmeasured diagnosis that influenced a person’s PLE estimate, then the effect of the unmeasured diagnosis would be attributed to the self-rated health measure. The variables included in the current analysis were selected based on being either established risk factors for health outcomes or low PLE in older adults, or, because they could plausibly be associated with expectations of longevity based on a biopsychosocial model of PLE [10]. A more readily interpretable question about PLE, rather than the probability-based measure used in the ELSA, could be a measure of what age the respondent thinks they live to.

The categorization of the PLE variable resulted in some loss of information on this variable, although the categorization was essential to reduce focal point bias caused by measurement error in the variable. Although the categorization resulted in lower sensitivity to detect associations of small magnitude, the prevalence ratios are estimated precisely with the large sample size of this study. Our PLE measure has been associated with actual mortality risk and prospective health behaviors such as uptake of cancer screening [3,13,14], and the predictors of PLE identified in this study are consistent with strong risk factors for 10-year all-cause mortality in the ELSA study population [29], underscoring the validity of our PLE measure. Overall, our study adds empirical evidence to support some aspects of Griffin’s original model, and builds upon it by including additional psychological factors related to quality of life. In order to refine understanding of how mental models of longevity are actually formed and maintained, in-depth qualitative research that directly asks participants about how they develop their expectations of their future longevity would be useful.

Another important area for future research is on the longitudinal and possibly bidirectional relationships between the biopsychosocial predictors under study here and PLE. Longitudinal study designs can help establish the temporality of associations, as well as the duration, timing in life, potential lag periods, and ‘dose’ of ‘exposure’ to any of the predictors under study here and PLE. For example, we identified that engaging in any moderate-to-vigorous physical activity (MVPA) was positively associated with PLE. The lifetime duration of MVPA, changes to MVPA, and the typical frequency and types of MVPA that may be associated with PLE over time are unknown. It might take a long duration of habitual MVPA to potentially influence mental models of PLE, whereas, for example, a diagnosis of a chronic disease such as cancer may affect one’s PLE estimate very quickly. The amount of theory and empirical evidence to build rationale for the expected nature of longitudinal influences on PLE is growing, with this study making a contribution to this area. Future research could utilize longitudinal models to assess the time-varying predictors that may influence within-person changes in PLE during aging.

Finally, ethnic differences in PLE could not be investigated, as only 3% of the sample reported belonging to a non-white ethnicity [26]. Although a total of 15% of data was missing, less than 5% of observations was missing for any one variable that had missing data. Respondents with any missing data were more likely to be older, to be male, to have worse health behaviors, to have lower CASP-19 scores, and lower self-rated health than those with complete data. As these factors were associated with low PLE in the final model, our effect estimates are likely underestimates of the true associations with PLE. The prevalence of low PLE is also an underestimate in our study due to missing data, and may be higher in the UK general population in reality. To account for any potential bias introduced by differential non-responses to the study, we used iteratively calculated population-based weights that were developed using the 2001 UK Census data [42]. The weights iteratively accounted for non-response to all waves of the ELSA to ensure that all estimated RRs were representative of the target general UK population. The main strength of this study is the rich data we had on a wide segment of the older English population, which allowed for the investigation of multiple sociodemographic, biomedical, behavioral, and psychological predictors of PLE using population-representative models.

## Conclusions

This study demonstrates that the predictors of people’s perceptions of their life expectancy are similar to actual risk factors for mortality. We newly identified that psychological indicators of well-being are associated with people’s expectations about their longevity: sense of control over life, life satisfaction, and perceived social standing. The consistency between the predictors of PLE in our study and known risk factors for mortality indicate that PLE may be a useful construct in health research. Our results may form a basis for future in-depth qualitative research that aims to understand how people develop their mental models of personal future longevity. Quantitative research of longitudinal design could also examine the stability of PLE estimates over time in response to various aspects of the aging process, as well as the possible birectional relationships between these factors and PLE in older adults. This is a rich area for future research. PLE might be a psychologically salient variable for health decision-making, behavior, and outcomes in later life that should be accounted for in future studies when appropriate, and investigated in its own right.

## Supporting information

S1 File Comparison of PLE with objective 10-year mortality risk. (PDF)
