# Supplementary material for: Biopsychosocial predictors of perceived life expectancy in a national sample of older men and women
Source: PLoS One. 2017 Dec 14;12(12):e0189245. doi: 10.1371/journal.pone.0189245 (PMC5730115; doi:10.1371/journal.pone.0189245)
Supplement: S1 File — (PDF) [file pone.0189245.s001.pdf]

S1 File

Comparison of PLE with objective 10-year mortality risk

PLE estimates in this study were consistent with objectively estimated risk of 10-year all-cause mortality, according to the ELSA Mortality Risk Index. The ELSA Mortality Risk Index is a valid and reliable objective indictor of 10-year all-cause mortality risk that assigns point scores for mortality risk based on current age, sex, the presence of comorbid conditions (cancer, lung disease, heart failure), smoking status, physical activity, and functional difficulties (walking 100 yards, pushing and pulling large objects, preparing a hot meal). Table 3 shows mean (SD) PLE scores according to 10-year mortality risk score. Mean PLE estimates steadily decreased with increasing point score on the ELSA Mortality Risk Index, ranging from a perceived 72.8 (SD: 19.7) percent chance of living another 10-15 years for those who scored 0 points on the index, to a perceived 40.8 (SD: 29.1) percent chance of living another 10-15 years for those who scored ≥11 points on the index (S1 Table).

S1 Table A. Mean PLE according to ELSA Mortality Index Point Score for 10-year All-Cause Mortality Risk, the English Longitudinal Study of Ageing, 2012-13 (n=6662)

Point Score	N	Mean PLE (SD)
0	451	72.8 (19.7)
1	809	71.2 (19.9)
2	870	68.7 (21.5)
3	993	67.6 (21.7)
4	676	64.5 (22.8)
5	633	63.2 (22.6)
6	582	58.9 (23.6)
7	465	54.8 (26.4)
8	466	48.9 (27.1)
9	273	47.9 (30.0)
10	277	44.7 (28.5)
≥11	166	40.8 (29.1)

S1 Table B. Comparison of characteristics of participants with any missing data vs. non-missing data, English Longitudinal Study of Ageing, 2012-13

**Characteristic (n)**	Any missing data (not in model) (N=1247/7909)	Complete data (included in model) (N=6662/7909)	p-value
Perceived life expectancy (n=7799)			<0.001
0-49%	285 (25%)	1208 (18%)	
50-74%	490 (43%)	2806 (42%)	
75-100%	362 (32%)	2648 (40%)	
**Sociodemographic factors**			
Educational attainment (n=7848)			<0.001
Higher education	301 (25%)	2310 (35%)	
Intermediate	483 (41%)	3043 (46%)	
No qualifications	402 (34%)	1309 (20%)	
Married or cohabiting (n=7909)	853 (68%)	5132 (77%)	<0.001
**Biomedical factors**			
Mean (SD) age (n=7909)	65.9 (8.0)	64.5 (7.4)	<0.001
Mean (SD) mother's age at death			
Mean (SD) father's age at death			
Male sex (n=7909)	509 (41%)	3006 (45%)	0.005
Depressive symptoms (n=7905)	365 (29%)	1436 (22%)	<0.001
Cardiovascular disease (n=7909)	134 (11%)	605 (9%)	0.06
Cancer (n=7909)	37 (3%)	242 (4%)	0.24
Diabetes (n=7909)	154 (12%)	668 (10%)	0.01
Chronic lung disease (n=7909)	70 (6%)	279 (4%)	0.02
**Health behaviors**			
Current smoking (n=7909)	204 (16%)	785 (12%)	<0.001
Alcohol consumption (n=7909)			<0.001
Abstainer	292 (23%)	777 (12%)	
Not excessive	757 (61%)	4420 (66%)	
Excessive	198 (16%)	1465 (22%)	
Fruit & vegetable intake <5 per day (n=7909)	617 (50%)	2746 (41%)	<0.001
Weekly physical activity (n=7909)			<0.001
None or mild	334 (27%)	1166 (18%)	
Moderate-to-vigorous	913 (73%)	5496 (83%)	
**Psychological factors**			
Mean (SD) loneliness (n=7774)	4.1 (1.2)	4.0 (1.2)	0.003
Mean (SD) control (n=7670)	7.5 (2.6)	8.0 (2.4)	<0.001
Mean (SD) autonomy (n=7686)	9.8 (2.9)	10.2 (2.7)	<0.001
Mean (SD) life satisfaction (n=7735)	9.3 (3.4)	10.1 (3.1)	<0.001
Mean (SD) pleasure (n=7733)	12.8 (2.7)	13.1 (2.3)	<0.001
Mean (SD) perceived social status (n=7517)	54.4 (18.4)	59.1 (17.2)	<0.001
Self-rated health			<0.001
Poor	136 (11%)	402 (6%)	
Fair	284 (23%)	1128 (17%)	
Good	399 (32%)	2118 (32%)	
Very good	309 (25%)	2097 (31%)	
Excellent	117 (9%)	917 (14%)	

S1 Table C. Correlations between study variables (n=6662)

	Age	Mother	Father	Sex	Educ	Married	Dep.	CVD	Ca.	Diab.	Lung	Smoke
Age	1.00	-	-	-	-	-	-		-	-	-	-
Mother	**0.10**	1.00	-	-	-	-	-		-	-	-	-
Father	**-0.06**	**0.09**	1.00	-	-	-	-		-	-	-	-
Sex	**0.04**	0.00	0.01	1.00	-	-	-		-	-	-	-
Educ.	**0.13**	**-0.09**	**-0.08**	**0.14**	1.00	-	-		-	-	-	-
Married	**0.15**	-0.01	**-0.04**	**0.14**	**0.10**	1.00	-		-	-	-	-
Depress	0.01	**-0.06**	-0.02	**0.11**	**0.14**	**0.13**	1.00		-	-	-	-
CVD	**0.18**	**-0.03**	**-0.07**	**0.08**	**0.07**	**0.06**	**0.12**	1.00				
Cancer	**0.04**	0.01	-0.02	**0.03**	0.02	0.01	**0.03**	0.00	1.00	-	-	-
Diabetes	**0.10**	**-0.03**	**-0.03**	**0.08**	**0.07**	**0.03**	**0.09**	**0.15**	0.01	1.00	-	-
Lung dx	**0.06**	**-0.06**	**-0.04**	0.01	**0.09**	**0.08**	**0.12**	**0.07**	**0.02**	**0.05**	1.00	-
Smoking	**-0.10**	**-0.07**	**-0.03**	0.00	**0.12**	**0.10**	**0.09**	**0.04**	0.00	0.00	**0.11**	1.00
MVPA	**-0.10**	**0.08**	**0.06**	**0.05**	**0.18**	**0.11**	**0.23**	**0.16**	**0.04**	**0.13**	**0.15**	**0.12**
F&V	**0.05**	**0.03**	0.02	**0.09**	**0.09**	**0.05**	**0.06**	**0.03**	0.00	**0.02**	**0.05**	**0.15**
Alc.	0.00	**0.07**	0.02	**0.14**	**-0.17**	**0.12**	**0.15**	**0.09**	0.02	**0.12**	**0.04**	**0.06**
Lonely	-0.02	**-0.03**	-0.02	**0.09**	**0.09**	**0.27**	**0.34**	**0.08**	0.02	**0.06**	**0.07**	**0.08**
C	**-0.12**	**0.03**	**0.03**	0.01	**-0.13**	**0.10**	**0.37**	**0.14**	**0.04**	**0.10**	**0.09**	**0.08**
A	0.06	**0.05**	0.02	0.01	**-0.09**	**0.03**	**0.32**	**0.12**	**0.04**	**0.10**	**0.09**	**0.09**
S	**-0.07**	**0.06**	**0.04**	0.01	**-0.17**	**0.16**	**0.36**	**0.14**	0.03	**0.11**	**0.13**	**0.12**
P	**0.05**	**0.04**	0.02	**0.04**	**-0.09**	**0.14**	**0.29**	**0.06**	0.01	**0.06**	**0.07**	**0.11**
Ladder	-0.02	**0.10**	**0.07**	**0.04**	**-0.30**	**-0.17**	**-0.26**	**-0.08**	0.00	**-0.09**	**-0.08**	**-0.14**
SRH	**-0.14**	**0.08**	**0.08**	0.03	**-0.20**	**0.13**	**0.39**	**0.26**	**0.11**	**0.24**	**0.24**	**0.13**

Note: Educ. = education; Dep. = depressive symptoms; Ca. = cancer; Diab. = diabetes; Lung dx = lung disease; MVPA = moderate-to-vigorous physical activity; F&V = fruit and vegetable servings; C = control; A = autonomy; S = life satisfaction; P = pleasure; Ladder = perceived social standing; SRH = self-rated health

Note: Bolded correlation coefficients are statistically significant with $p<0.05$

Note: Association types are organized by color. Purple = Spearman's correlation coefficient; Yellow = point biserial correlation coefficient; Green = phi correlation coefficient; Blue = Cramer's V (range is from 0 to 1 and does not indicate the direction of association; statistical significance is determined by the chi-squared test for the contingency table)

S1 Table C Continued. Correlations between all study variables (n=6662)

	MVPA	F&V	Alc.	Lonely	C	A	S	P	Ladder	SRH
Age	-	-	-	-	-	-	-	-	-	-
Mother	-	-	-	-	-	-	-	-	-	-
Father	-	-	-	-	-	-	-	-	-	-
Sex	-	-	-	-	-	-	-	-	-	-
Educ.	-	-	-	-	-	-	-	-	-	-
Married	-	-	-	-	-	-	-	-	-	-
Depress	-	-	-	-	-	-	-	-	-	-
CVD										
Cancer	-	-	-	-	-	-	-	-	-	-
Diabetes	-	-	-	-	-	-	-	-	-	-
Lung dx	-	-	-	-	-	-	-	-	-	-
Smoking	-	-	-	-	-	-	-	-	-	-
MVPA	1.00	-	-	-	-	-	-	-	-	-
F&V	**0.10**	1.00	-	-	-	-	-	-	-	-
Alc.	**0.16**	**0.09**	1.00	-	-	-	-	-	-	-
Lonely	**0.12**	**0.08**	**-0.11**	1.00	-	-	-	-	-	-
C	**0.21**	**0.09**	**0.12**	**-0.50**	1.00	-	-	-	-	-
A	**0.21**	**0.08**	**0.13**	**-0.33**	**0.61**	1.00	-	-	-	-
S	**0.26**	**0.14**	**0.12**	**-0.40**	**0.60**	**0.54**	1.00	-	-	-
P	**0.14**	**0.14**	**0.09**	**-0.43**	**0.47**	**0.41**	**0.65**	1.00	-	-
Ladder	**0.20**	**0.10**	**0.19**	**-0.23**	**0.33**	**0.35**	**0.40**	**0.30**	1.00	-
SRH	**0.41**	**0.11**	**0.15**	**-0.21**	**0.41**	**0.40**	**0.46**	**0.28**	**0.28**	1.00

Note: Educ. = education; Dep. = depressive symptoms; Ca. = cancer; Diab. = diabetes; Lung dx = lung disease; MVPA = moderate-to-vigorous physical activity; F&V = fruit and vegetable servings; C = control; A = autonomy; S = life satisfaction; P = pleasure; Ladder = perceived social standing; SRH = self-rated health

Note: Bolded correlation coefficients are statistically significant with $p<0.05$

Note: Association types are organized by color. Purple = Spearman's correlation coefficient; Yellow = point biserial correlation coefficient; Green = phi correlation coefficient; Blue = Cramer's V (range is from 0 to 1 and does not indicate the direction of association; statistical significance is determined by the chi-squared test for the contingency table)

S1 Table D. Collinearity diagnostics (n=6662)

Variable	Tolerance	VIF
Age	0.83	1.20
Mother’s age at death	0.97	1.03
Father’s age at death	0.98	1.02
Sex	0.90	1.11
Education	0.83	1.20
Marital status	0.85	1.18
Depressive symptoms	0.76	1.32
CVD	0.89	1.12
Cancer	0.98	1.02
Diabetes	0.92	1.08
Lung disease	0.94	1.07
Smoking	0.93	1.08
MVPA	0.83	1.20
Fruit & vegetable intake	0.93	1.07
Alcohol consumption	0.91	1.10
Loneliness	0.63	1.56
Control	0.45	2.23
Autonomy	0.52	1.92
Life satisfaction	0.41	2.47
Pleasure	0.52	1.92
Perceived social status	0.72	1.39
Self-rated health	0.59	1.71

S1 Table E. Population-weighted and adjusted prevalence ratios (PRs) for medium and low vs. high perceived life expectancy (PLE) associated with sociodemographic, biomedical, behavioral, and psychosocial factors, the English Longitudinal Study of Ageing, 2012-13, ages <65 years (n=3430)

Characteristic	PR[a] Medium PLE	95% CI	PR[b] Low PLE	95% CI
**Sociodemographic factors**				
Intermediate education (vs. higher)	1.03	(0.92, 1.15)	1.30	(0.97, 1.74)
No educational qualifications (vs. higher)	1.14	(0.99, 1.31)	1.48	(1.05, 2.07)
Single marital status	1.07	(0.90, 1.29)	0.94	(0.68, 1.27)
**Biomedical factors**				
Current age (per year)	1.01	(1.00, 1.03)	1.02	(0.99, 1.05)
Mother's age at death (per 10 years)	0.96	(0.92, 0.99)	0.91	(0.85, 0.98)
Father's age at death (per 10 years)	0.97	(0.94, 1.01)	0.85	(0.79, 0.92)
Male sex	1.00	(0.91, 1.11)	1.19	(0.97, 1.47)
Depressive symptoms	1.00	(0.88, 1.13)	0.98	(0.76, 1.28)
Cardiovascular disease	0.71	(0.56, 0.90)	0.89	(0.65, 1.23)
Cancer	1.46	(1.20, 1.77)	1.88	(1.32, 2.67)
Diabetes	1.11	(0.93, 1.32)	1.32	(0.99, 1.75)
Chronic lung condition	1.15	(0.91, 1.45)	1.33	(0.95, 1.86)
**Health behaviors**				
Current smoking	1.19	(1.05, 1.33)	1.53	(1.21, 1.93)
Any MVPA (vs. mild/no physical activity)	1.15	(1.01, 1.32)	0.95	(0.73, 1.24)
Less than five daily fruit or vegetable servings	1.09	(0.99, 1.20)	1.25	(0.99, 1.56)
Non-excessive alcohol drink (vs. abstainer)	0.92	(0.79, 1.06)	0.77	(0.57, 1.04)
Excessive alcohol drinker (vs. abstainer)	0.90	(0.75, 1.08)	0.86	(0.61, 1.21)
**Psychological factors**				
Loneliness[c]	0.95	(0.90, 1.00)	0.95	(0.86, 1.05)
Control[c]	0.98	(0.95, 1.01)	0.92	(0.86, 0.97)
Autonomy[c]	0.99	(0.94, 1.06)	0.80	(0.68, 0.94)
Pleasure[c]	1.01	(0.92, 1.10)	1.13	(0.92, 1.38)
Life satisfaction[c]	0.89	(0.83, 0.96)	0.96	(0.80, 1.16)
Perceived social status (per 10 points)	0.98	(0.95, 1.01)	0.91	(0.85, 0.96)
Self-rated health[c]	0.89	(0.84, 0.94)	0.78	(0.68, 0.90)

[a]PR predicts medium (50-74%) vs. high (75-100%) PLE

[b]PR predicts low (0-49%) vs. high (75-100%) PLE

[c]PR is for increasing linear trend from lowest to highest quartiles of the variable

S1 Table F. Population-weighted and adjusted prevalence ratios (PRs) for medium and low vs. high perceived life expectancy (PLE) associated with sociodemographic, biomedical, behavioral, and psychosocial factors, the English Longitudinal Study of Ageing, 2012-13, ages ≥65 years (n=3232)

Characteristic	PR[a] Medium PLE	95% CI	PR[b] Low PLE	95% CI
**Sociodemographic factors**				
Intermediate education (vs. higher)	1.00	(0.92, 1.10)	0.90	(0.80, 1.02)
No educational qualifications (vs. higher)	0.95	(0.86, 1.06)	0.94	(0.83, 1.08)
Single marital status	1.00	(0.83, 1.22)	0.95	(0.72, 1.25)
**Biomedical factors**				
Current age (per year)	1.01	(1.01, 1.02)	1.07	(1.06, 1.08)
Mother's age at death (per 10 years)	0.94	(0.92, 0.97)	0.91	(0.88, 0.94)
Father's age at death (per 10 years)	0.98	(0.95, 1.00)	0.94	(0.91, 0.97)
Male sex	1.03	(0.96, 1.12)	1.07	(0.97, 1.19)
Depressive symptoms	0.96	(0.86, 1.06)	0.93	(0.83, 1.04)
Cardiovascular disease	0.98	(0.88, 1.09)	1.00	(0.87, 1.23)
Cancer	1.01	(0.83, 1.22)	1.04	(0.87, 1.23)
Diabetes	0.95	(0.84, 1.07)	1.12	(0.99, 1.26)
Chronic lung condition	1.02	(0.87, 1.20)	1.10	(0.94, 1.28)
**Health behaviors**				
Current smoking	1.03	(0.90, 1.17)	1.18	(1.02, 1.36)
Any MVPA (vs. mild/no physical activity)	1.12	(1.00, 1.24)	1.23	(1.09, 1.38)
Less than five daily fruit or vegetable servings	1.04	(0.96, 1.12)	0.99	(0.90, 1.10)
Non-excessive alcohol drink (vs. abstainer)	1.06	(0.95, 1.19)	1.09	(0.96, 1.25)
Excessive alcohol drinker (vs. abstainer)	0.97	(0.85, 1.10)	1.04	(0.89, 1.23)
**Psychological factors**				
Loneliness[c]	0.97	(0.93, 1.01)	0.93	(0.89, 0.98)
Control[c]	0.99	(0.97, 1.01)	0.98	(0.96, 1.01)
Autonomy[c]	1.03	(0.98, 1.08)	1.01	(0.95, 1.08)
Pleasure[c]	0.98	(0.92, 1.03)	0.95	(0.88, 1.02)
Life satisfaction[c]	0.94	(0.89, 0.98)	0.88	(0.82, 0.94)
Perceived social status (per 10 points)	0.98	(0.96, 1.01)	0.93	(0.90, 0.96)
Self-rated health[c]	0.91	(0.87, 0.95)	0.78	(0.73, 0.82)

[a]PR predicts medium (50-74%) vs. high (75-100%) PLE

[b]PR predicts low (0-49%) vs. high (75-100%) PLE

[c]PR is for increasing linear trend from lowest to highest quartiles of the variable
